# Acute kidney injury is associated with impaired cognition and chronic kidney disease in a prospective cohort of children with severe malaria

**DOI:** 10.1186/s12916-019-1332-7

**Published:** 2019-05-21

**Authors:** Andrea L. Conroy, Robert O. Opoka, Paul Bangirana, Richard Idro, John M. Ssenkusu, Dibyadyuti Datta, James S. Hodges, Catherine Morgan, Chandy C. John

**Affiliations:** 1 Ryan White Center for Pediatric Infectious Disease and Global Health, Indiana University School of Medicine, 1044 W. Walnut St., Indianapolis, IN 46202 USA; 2 Department of Paediatrics and Child Health, Makerere University College of Health Sciences, Kampala, Uganda; 3 Department of Psychiatry, Makerere University College of Health Sciences, Kampala, Uganda; 4 Department of Epidemiology and Biostatistics, Makerere University School of Public Health, Kampala, Uganda; 5 Division of Biostatistics, School of Public Health, University of Minnesota, Minneapolis, USA; 6 Division of Pediatric Nephrology, University of Alberta, Edmonton, Canada; 7 Division of Global Pediatrics, University of Minnesota Medical School, Minneapolis, USA

**Keywords:** Acute kidney injury, Malaria, Child, Cognition, Mortality, Chronic kidney disease

## Abstract

**Background:**

Acute kidney injury (AKI) is a recognized complication of pediatric severe malaria, but its long-term consequences are unknown.

**Methods:**

Ugandan children with cerebral malaria (CM, *n* = 260) and severe malaria anemia (SMA, *n* = 219) or community children (CC, *n* = 173) between 1.5 and 12 years of age were enrolled in a prospective cohort study. Kidney Disease: Improving Global Outcomes (KDIGO) criteria were used to retrospectively define AKI and chronic kidney disease (CKD). Cognitive testing was conducted using the Mullen Scales of Early Learning in children < 5 and Kaufman Assessment Battery for Children (K-ABC) second edition in children ≥ 5 years of age.

**Results:**

The prevalence of AKI was 35.1%, ranging from 25.1% in SMA to 43.5% in CM. In-hospital mortality was 11.9% in AKI compared to 4.2% in children without AKI (*p* = 0.001), and post-discharge mortality was 4.7% in AKI compared to 1.3% in children without AKI (*p* = 0.030) corresponding to an all-cause adjusted hazard ratio of 2.30 (95% CI 1.21, 4.35). AKI was a risk factor for short- and long-term neurocognitive impairment. At 1 week post-discharge, the frequency of neurocognitive impairment was 37.3% in AKI compared to 13.5% in children without AKI (adjusted odds ratio (aOR) 2.31 [95% CI 1.32, 4.04]); at 1-year follow-up, it was 13.3% in AKI compared to 3.4% in children without AKI (aOR 2.48 [95% CI 1.01, 6.10]), and at 2-year follow-up, it was 13.0% in AKI compared to 3.4% in children without AKI (aOR 3.03 [95% CI 1.22, 7.58]). AKI was a risk factor for CKD at 1-year follow-up: 7.6% of children with severe malaria-associated AKI had CKD at follow-up compared to 2.8% of children without AKI (*p* = 0.038) corresponding to an OR of 2.81 (95% CI 1.02, 7.73). The presenting etiology of AKI was consistent with prerenal azotemia, and lactate dehydrogenase as a marker of intravascular hemolysis was an independent risk factor for AKI in CM and SMA (*p* < 0.0001). In CM, AKI was associated with the presence and severity of retinopathy (*p* < 0.05) and increased cerebrospinal fluid albumin suggestive of blood-brain barrier disruption.

**Conclusions:**

AKI is a risk factor for long-term neurocognitive impairment and CKD in pediatric severe malaria.

**Electronic supplementary material:**

The online version of this article (10.1186/s12916-019-1332-7) contains supplementary material, which is available to authorized users.

## Background

Infection with *Plasmodium falciparum* is a significant cause of global morbidity and mortality: an estimated 219 million cases of malaria were reported in 2017 with 92% of estimated cases occurring in sub-Saharan Africa [1]. Severe malaria is also a leading cause of acquired neurodisability in African children [2]. Clinical risk factors described to date for neurocognitive impairment in severe malaria are acute neurologic manifestations, e.g., duration of coma and number of seizures [2–6].

Although children with severe malaria may present with signs suggesting a focal insult, multi-organ dysfunction is common [7–9]. Acute kidney injury (AKI) is a common complication of pediatric severe malaria [9, 10] associated with mortality [9–13] and neurologic deficits in survivors [10]. In a meta-analysis of predictors of mortality in African children with severe malaria, AKI was the strongest predictor of death with an odds ratio of 5.96 (95% confidence interval (CI) 2.93 to 12.11) [14]. AKI is an established clinical risk factor for chronic kidney disease (CKD) in adults [15], but information on long-term renal recovery after AKI in pediatric populations is lacking. In particular, there are no data on whether AKI in severe malaria is a risk factor for CKD.

In this prospective cohort study, we evaluated the prevalence of AKI in pediatric severe malaria at admission and investigated the relationship between AKI and clinical and renal recovery, and also with long-term neurocognitive functioning.

## Methods

### Study participants

The study was performed at Mulago National Referral Hospital in Uganda from 2008 to 2015, enrolling children 18 months to 12 years of age as described [4] (Additional file 1). All children with *P. falciparum* on blood smear who met the inclusion criteria for cerebral malaria (CM) and severe malarial anemia (SMA) were enrolled. Children with CM had a coma with no other identifiable cause ruling out meningitis, a prolonged postictal state, or hypoglycemia-associated coma reversed by a glucose infusion. Children with SMA had hemoglobin level ≤ 5 g/dL. Children with CM and severe anemia were classified as CM. Age-matched community children (CC) were recruited from the nuclear family, extended family, or household area of children with severe malaria (CM or SMA). Exclusion criteria included prior coma, head trauma, hospitalization for malnutrition, cerebral palsy, or known chronic illness requiring medical care or causing developmental delay.

Children were managed according to the Uganda Clinical Guidelines at the time of the study, including intravenous infusion of 10 mg/kg quinine hydrochloride in 5–10 mL/kg of 5% glucose over a 4-h period for the treatment of severe malaria, repeated every 8 h until the child could take oral medication (quinine or artemether-lumefantrine). Towards the end of the study, the treatment shifted towards the use of parenteral artemisinin-based therapies for the treatment of severe malaria following the 2011 World Health Organization recommendation of injectable artesunate as the first-line treatment for severe malaria. Hypoglycemia was treated with a 1–2-mL/kg 25% dextrose bolus administered intravenously. Fluid resuscitation was managed conservatively according to local guidelines at the time of the study: a fluid bolus of 20 mL/kg of sodium chloride 0.9% intravenously over 1 h was given only for the treatment of shock (systolic blood pressure < 50 mmHg or absent peripheral pulse) with delayed capillary refill (> 2 s). Children without shock but with evidence of dehydration received maintenance intravenous fluids. Furosemide was administered to children with clinical evidence of congestive heart failure or lack of urine output over one or more 8-h shifts, after ruling out dehydration and shock, at a dose of 1 mg/kg up to a maximum of 4 mg/kg daily. Dialysis was not available on site at the time the study was conducted.

All children underwent a medical history and physical examination on enrollment. As a measure of disease severity, we evaluated the number of severe malaria criteria present (Additional file 1: Table S1, Methods). Emotional stimulation was assessed using age-appropriate versions of the Home Observation for the Measurement of the Environment (HOME) [4].

### Laboratory assessment

Peripheral blood smears were used to quantify parasite density using Giemsa staining with standard protocols. EDTA anticoagulated plasma was collected at admission and stored at − 80 °C until testing. Plasma PfHRP2 levels were measured to assess parasite biomass (Cellabs, Australia) [16]. Creatinine was tested on cryopreserved enrollment samples using a Beckman Coulter AU680 using the modified Jaffe method (Indiana University, Pathology Laboratory). Samples were sent in batches to the clinical laboratory for creatinine testing between 2012 and 2014, and 1-year follow-up samples were tested in 2016.

### Assessment of kidney function

Acute kidney injury (AKI) was defined retrospectively using Kidney Disease: Improving Global Outcomes (KDIGO) guidelines [17] based on a single admission creatinine level, with baseline creatinine estimated using CC as described in Additional file 1. AKI was defined as a 1.5-fold increase in creatinine over baseline and was staged: stage 1, 1.5–1.9× increase in creatinine over baseline; stage 2, 2.0–2.9× increase over baseline; and stage 3, ≥ 3.0× increase over baseline [17]. Chronic kidney disease (CKD) was defined as an eGFR< 90 mL/min/1.73 m^2^ using the Bedside Schwartz equation and categorized using KDIGO guidelines [18]. The AKI classification was repeated using an epidemiologic method to estimate baseline creatinine [19] (Additional file 1: Table S2). A comparative analysis is presented in Additional file 1.

### Neurocognitive assessment

Children were tested at enrollment (CC) or a week after discharge (CM, SMA) and at 1- and 2-year follow-up. Cognition in children less than 5 years of age was assessed by the Mullen Scales of Early Learning. Scores from fine motor, visual reception, receptive language, and expressive language scales were summed to give the early learning composite score, a measure of the overall cognitive ability. Cognitive ability in children 5 years of age and older was assessed by the Kaufman Assessment Battery for Children second edition (KABC-II). The composite score for the mental processing index was used to assess the child’s cognitive ability. Both tests have been validated previously in Ugandan children [2, 4]. Neurocognitive impairment was defined as the presence of a gross deficit on neurologic exam or an age-adjusted cognitive *z* score more than two standard deviations below the mean.

### Statistical analyses

Age-adjusted *z* scores for cognition were created using the community children [2, 3]. Unadjusted comparisons for continuous and categorical measures used the Wilcoxon rank-sum test or Kruskal-Wallis test and Pearson’s *χ*^2^ or Fisher’s exact test, respectively. Logistic regression was used to estimate factors’ association with AKI and to test the association between AKI, retinopathy, and neurocognitive outcomes. Ordinal logistic regression was used to assess the relationship between AKI and the ordinal categories of retinopathy. Cox proportional hazards regression was used to evaluate the association between AKI and death. Logistic regression was used to evaluate the association between AKI and cerebrospinal fluid albumin. Adjusters were included in multivariable analyses if they had *p* < 0.10 in a bivariate analysis or an a priori hypothesized relationship with the outcome. Holm’s correction was used to adjust for multiple comparisons. Analyses were done using Stata v14.0 (StataCorp. 2015).

### Role of the funding source

The funders had no role in the study design, analysis, or decision to publish.

## Results

**Fig. 1:**
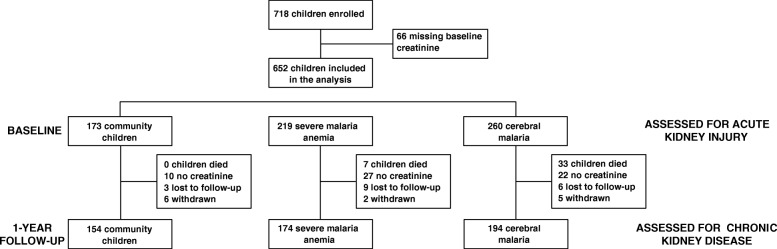
Flow chart of the study population. Children with a creatinine on study enrollment were included in the study and had their kidney function re-assessed at 1-year follow-up

In total, 652 children with enrollment creatinine values were included in the study (Fig. 1). The demographic characteristics of the population are presented in Table 1. The prevalence of AKI was 35.1% overall: 43.5% of children with CM and 25.1% of children with SMA. Children with CM had a more severe AKI than children with SMA (CM: 23.5% stage 1 (*n* = 61), 12.7% stage 2 (*n* = 33), 7.3% stage 3 (*n* = 19); SMA: 16.0% stage 1 (*n* = 35), 7.3% stage 2 (*n* = 16), 1.8% stage 3 (*n* = 4), *p* < 0.0001). Children with CM had a 2.37-fold (95% CI 1.38, 4.08) increase in the odds of severe AKI (AKI stage 2 or 3) compared to children with SMA (*p* = 0.010). The prevalence of AKI was similar in children treated with quinine, 34.7% (*n* = 133), vs. those treated with parenteral artemisinin derivatives, 36.0% (*n* = 55), *p* = 0.78.

**Table 1:** Demographic and laboratory characteristics of study children

Characteristic	CC (*n* = 173)	SMA (*n* = 219)	CM (*n* = 260)	*p* value
Demographics				
Age, median (IQR), years	3.6 (2.6, 4.6)	2.9 (2.1, 4.5)	3.5 (2.5, 4.9)	0.0002
Female sex, (%) no.	94 (54.3)	86 (39.3)	107 (41.2)	0.006
Weight-for-age *z* score, median (IQR)	− 1.0 (− 1.5, − 0.3)	− 1.5 (− 2.2, − 0.7)	− 1.2 (− 1.9, − 0.5)	0.0001
Height-for-age *z* score, median (IQR)	− 1.1 (− 1.7, − 0.4)	− 1.0 (− 1.9, − 0.3)	− 0.6 (− 1.9, − 0.5)	0.002
Socioeconomic status score, median (IQR)^1^	9 (8, 12)	9 (7, 11)	9 (8, 11)	0.355
Home environment *z* score, median (IQR)^1^	0.12 (− 0.71, 0.85)	− 0.05 (− 0.71, 0.67)	− 0.02 (− 0.71, 0.71)	0.523
Maternal education level, no. (%)^1^				
Primary 6 or lower	47 (27.2)	88 (38.6)	80 (36.7)	0.348
Primary 7	42 (24.3)	47 (20.6)	45 (20.6)	
Secondary or higher	74 (42.8)	80 (35.1)	83 (38.1)	
Not known	10 (5.8)	13 (5.7)	10 (4.6)	
Paternal education level, no. (%)^1^				
Primary 6 or lower	24 (13.9)	50 (22.9)	38 (16.7)	0.076
Primary 7	33 (19.1)	31 (14.2)	40 (17.5)	
Secondary or higher	90 (52.0)	98 (45.0)	98 (43.0)	
Not known	26 (15.0)	39 (17.9)	52 (22.8)	
Child any education, no. (%)^1^	72 (41.9)	56 (26.3)	85 (38.1)	0.008
Laboratory characteristics^2^				SMA vs. CM
Hemoglobin, g/dL	11.8 (11.0, 12.6)	3.9 (3.2, 4.5)	6.8 (5.1, 8.7)	< 0.0001
Glucose, mmol/L	–	6.4 (4.7, 8.2)	6.6 (4.9, 8.9)	0.162
Lactate, mmol/L	–	4.8 (3.0, 8.0)	3.8 (2.2, 6.7)	0.002
WBC, × 10^3^/μL	8.6 (7.2, 10.6)	11.5 (8.2, 16.2)	9.4 (7.2, 13.9)	0.0001
Platelet, ×10^3^/μL	383 (289, 449)	149 (91, 225)	60 (34, 111)	< 0.0001
Total bilirubin, mg/dL	0.2 (0.1, 0.3)	1.3 (0.7, 2.1)	1.6 (0.9. 2.7)	0.004
Lactate dehydrogenase (LDH), U/L	162 (232, 311)	762 (621, 990)	829 (630, 1116)	0.069
Plasma albumin, g/dL	3.7 (3.4, 3.9)	2.6 (2.3, 3.0)	2.6 (2.4, 2.9)	0.898
Sodium, mmol/L	137 (135, 138)	134 (132, 136)	132 (128, 136)	< 0.0001
Peripheral parasite density, parasites/uL	0 (0, 0)	34,970 (10,085, 134,925)	48,420 (10,830, 282,400)	0.0286
Plasma PfHRP2, ng/mL	5 (5, 118)	944 (367, 2790)	2828 (1024, 5546)	< 0.0001
Creatinine, mg/dL	0.30 (0.24, 0.35)	0.35 (0.28, 0.46)	0.42 (0.31, 0.55)	< 0.0001
BUN, mg/dL	7 (5, 9)	13 (9, 20)	17 (12, 25)	< 0.0001

Continuous measures presented as median (interquartile range) unless otherwise indicated. Continuous measures compared using the Kruskal-Wallis test. Count measures compared using Pearson’s chi-square or Fisher’s exact, as appropriate

^1^Assessed on survivors (*n* = 218 in SMA, *n* = 228 in CM)

^2^Data on laboratory characteristics presented for all children, as available, but analyzed using Wilcoxon rank-sum test comparing the differences between SMA and CM

### Fluid management, kidney function, and hydration status

The distribution of supportive care and fluid management by severe malaria group and AKI status is shown in Table 2. Blood products were commonly administered with 368 children (76.8%) receiving a transfusion during hospitalization. Details on the type of transfusion were available for 107 children, with 104 (96.3%) receiving packed red blood cells. Seventy-three (15.2%) children enrolled in the study received intravenous fluids (including 0.9% saline, Ringer’s lactate, or albumin) in addition to parenteral antimalarial therapy. No children fulfilled the criteria for an intravenous fluid bolus. Children with SMA and AKI were more likely to receive a dextrose bolus, and children with CM and AKI were more likely to receive intravenous fluids, a blood transfusion, and furosemide (adjusted *p* < 0.05, following Holm’s correction for multiple comparisons; Table 2).

### Mortality

Overall, the mortality was 9.2% with 44 deaths during the 2-year study period: 33 (75%) occurring during hospitalization and 11 (25%) occurring during follow-up. With the exception of 1 child with SMA who died of hypoglycemia, all in-hospital study deaths occurred in children with CM and were related to respiratory failure. Four (12.1%) children had clinical signs of cerebral edema identified as a contributing cause of death. Children who died were more likely to have received intravenous fluids during admission (33.3%, *n* = 11/33) compared to survivors (13.9%, *n* = 62/446) (*p* = 0.003) with the effect stronger in children without AKI (OR (95% CI), 3.69 (1.07, 12.68), *p* = 0.038) compared to children with AKI (OR (95% CI), 2.12 (0.78, 5.77), *p* = 0.14). Most follow-up deaths (7 of 11) occurred in the first 6 months of follow-up, and all follow-up deaths occurred in children who had severe anemia at the time of study enrollment. Details on the cause of death in follow-up were limited as several deaths occurred out of the hospital, but a history of fever, anemia, and malaria was common.

### Association between AKI and clinical recovery

**Fig. 2:**
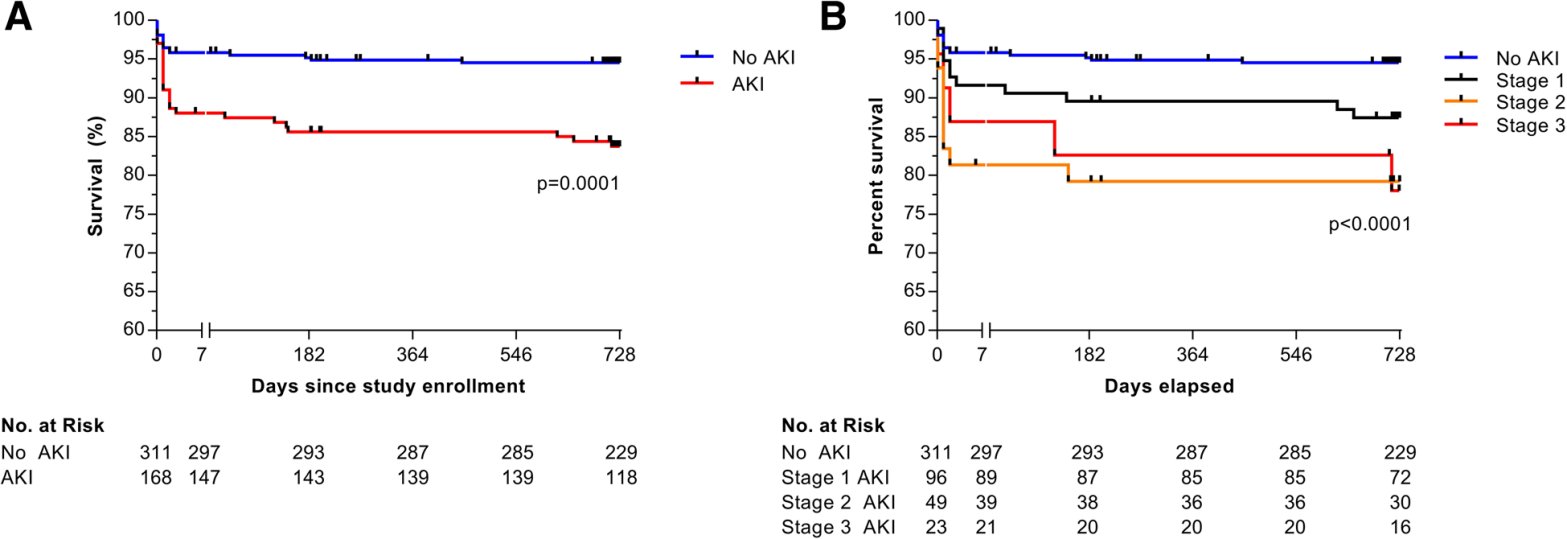
Mortality associated with acute kidney injury and across stages of AKI over 2-year follow-up. Kaplan-Meier plots showing 2-year survival in children with severe malaria based on the presence of KDIGO-defined acute kidney injury (AKI) status at admission (**a**) or the severity of AKI based on KDIGO-defined AKI stage (**b**). The break in the horizontal axis separates the first 7 days of follow-up (where the majority of in-hospital deaths occur) from the period of outpatient follow-up. Testing used the log-rank Mantel-Cox test in (**a**) and log-rank test for trend across stages of AKI (**b**)

Consistent with previous reports, AKI was associated with increased mortality (Fig. 2). In-hospital mortality was 11.9% (*n* = 20/168) for children with AKI compared to 4.2% (*n* = 13/311) in children without AKI (*p* = 0.001), and post-discharge mortality was 4.7% (*n* = 7/148) in children with AKI compared to 1.3% (*n* = 4/298) in children without AKI (*p* = 0.030) (Additional file 1: Tables S10-S11). Overall, 60.6% of in-hospital deaths and 63.6% of post-discharge deaths occurred in children with AKI. In total, 326 (68.1%) children were treated with intravenous quinine, and 153 (31.9%) were treated with parenteral artemisinin derivatives. In children receiving quinine, 14 (12.4%) children with AKI died compared to 13 (6.1%) children without AKI (*p* = 0.050). In children receiving artemisinin derivatives, 6 (10.9%) of those with AKI died compared to 0 (0.0%) children without AKI (*p* = 0.001). From admission to 2-year follow-up, AKI had an adjusted hazards ratio (aHR) for the death of 2.30 (95% CI 1.21, 4.35) following adjustment for age, sex, hemoglobin, disease severity, and parenteral antimalarial treatment (quinine vs. artemisinin derivative) (*p* = 0.0109).

Among the CM survivors, AKI was associated with prolonged fever clearance time (*p* < 0.0001), coma duration (*p* < 0.0001), and length of hospitalization (*p* = 0.0005) (Additional file 1: Table S10-S11). Among the SMA survivors, recovery times were not associated with AKI status.

### Association between severe malaria, AKI, and chronic kidney disease 1 year later

In children with a history of severe malaria, AKI was a risk factor for CKD with 9 (7.6%) children with AKI on admission having CKD at 1 year follow-up (7.1% in SMA, 7.8% in CM) compared to 7 (2.8%) children without AKI (odds ratio for CKD 2.81 [95% CI 1.02, 7.73]). In children with a history of CM, severe AKI was associated with a 4.08-fold (95% CI 1.03, 16.09) increase in the odds of CKD. Thirteen children with CKD (81.3%) were GFR category 2 (eGFR, 60–89 mL/min/1.73 m^2^) and 3 (18.8%) category 3 (eGFR, 30–59). The prevalence of CKD in children treated with quinine was 3.1% (*n* = 8/257) compared to 7.3% (*n* = 8/109) in children treated with artemisinin derivatives (*p* = 0.071). When stratifying by AKI status at enrollment, the prevalence of CKD without AKI was 1.7% (*n* = 3/173) in children treated with quinine vs. 5.4% (*n* = 4/74) in children treated with artemisinin derivatives (*p* = 0.202), and in children with AKI, the prevalence of CKD was 6.0% (*n* = 5/84) in children receiving quinine vs. 11.4% (*n* = 4/35) in children treated with artemisinin derivatives (*p* = 0.446). Children were screened for active illness prior to the blood draw, so the eGFR was unlikely to represent a repeat episode of AKI (detailed in Additional file 1: Methods).

### Association between AKI and neurocognitive outcomes

Neurologic deficits were common in CM survivors 1 week post-discharge (36.4%, *n* = 82) and consisted of speech difficulties (31.7%, *n* = 26), visual impairment (19.5%, *n* = 16), motor deficits (54.9%, *n* = 45), movement disorders (4.9%, *n* = 4), ataxia (47.6%, *n* = 39), and hyporeflexia or Babinski sign (32.9%, *n* = 27). Deficits persisted in only 3.1% of children (*n* = 7) at 2-year follow-up and were related to speech (71.4%) and movement disorders or motor deficits (85.7%). Overall, 53.3% (*n* = 48/90) of children with AKI had a neurologic deficit 1 week post-discharge compared to 25.2% (*n* = 34/135) of children without AKI (*p* < 0.0001). When stratifying by anti-malarial treatment, the association between AKI and neurologic deficit at discharge remained significant with OR (95% CI) of 3.31 (1.67, 6.56) in children treated with quinine (*p* = 0.0006) and 5.20 (1.62, 16.74) in children treated with artemisinin derivatives (*p* = 0.006). The association between AKI and neurologic deficits persisted at 2-year follow-up: 6.7% (*n* = 6/90) of children with a history of AKI had a neurologic deficit compared to 0.7% (*n* = 1/136) in children without a history of AKI (*p* = 0.017).

**Fig. 3:**
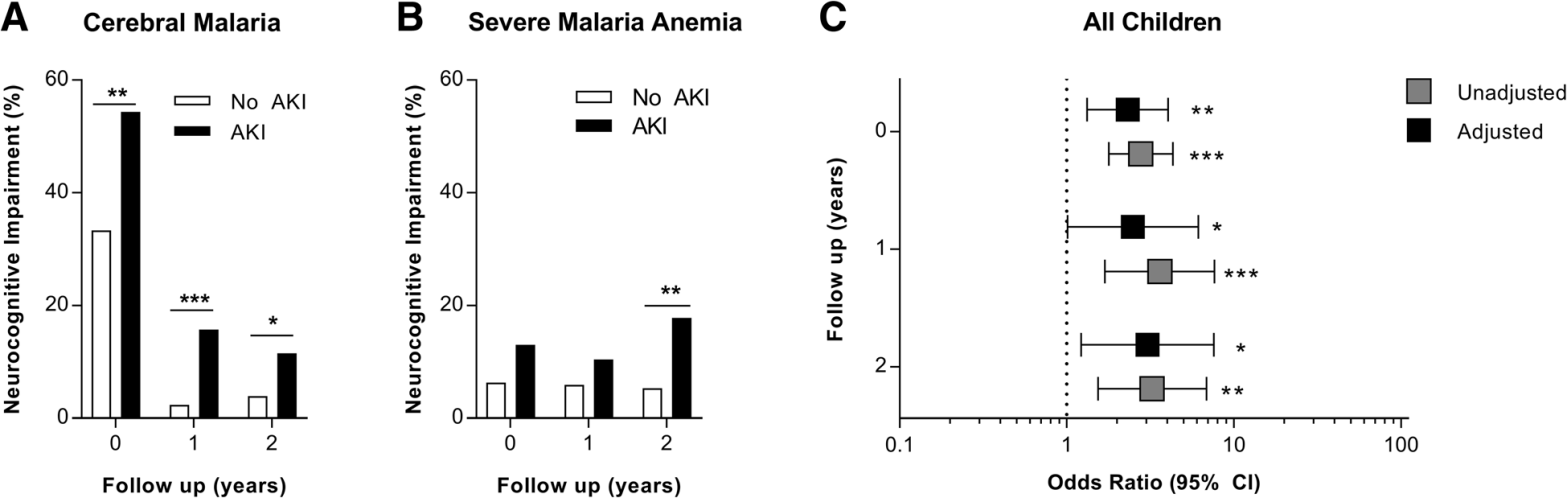
Association between acute kidney injury and neurocognitive recovery in children following severe malaria. Bar graphs showing the frequency of neurocognitive impairment in children with cerebral malaria (**a**) or severe malarial anemia (**b**). Neurocognitive impairment was defined as a gross deficit on the neurologic exam or an age-adjusted *z* score more than two standard deviations below the mean. Data analyzed using Pearson’s chi-square, *p < 0.01, **p < 0.01, ***p < 0.0001. **c**. Odds ratio of neurocognitive impairment (95% CI) associated with acute kidney injury from logistic regression models, 1 week post-discharge and 1- and 2-year follow-up. Multivariable-adjusted models included child age, sex, height- and weight-for-age *z* score, parental education, child schooling, an assessment of enrichment in the home environment, socioeconomic status, disease severity on presentation, the presence of coma, and number of seizures during hospitalization and parenteral antimalarial treatment (quinine vs. artemisinin derivative). *p < 0.05, **p < 0.01, ***p < 0.0001 following adjustment for multiple comparisons

As children surviving severe malaria are at risk of long-term neurocognitive impairment [5], we tested the association between AKI and neurocognition. AKI was associated with neurocognitive impairment 1 week post-discharge, with aOR (95% CI) of 2.31 (1.32, 4.04), and the effect persisted over time with aORs (95% CI) of 2.48 (1.01, 6.10) and 3.03 (1.22, 7.58) at 1- and 2-year follow-up, respectively (Fig. 3; Additional file 1: Tables S12-S13). The associations remained significant at 2-year follow-up after correction for multiple comparisons. Models were adjusted for demographic factors known to be associated with child development (age, sex, parental education, school attendance, socioeconomic status, enrichment in the home environment, and height- and weight-for-age *z* scores), a composite measure of disease severity, complications associated with impaired cognition (number of seizures, coma), and parenteral antimalarial treatment (quinine vs. artemisinin derivative).

### Etiology and pathophysiology of AKI in severe malaria

The majority of children with severe malaria had a BUN to creatinine ratio consistent with prerenal azotemia (CM, 96.5%; SMA, 89.6%). There was no association between hydration status (capillary refill time, sunken eyes, decreased skin turgidity, cool peripheries, systolic blood pressure, dry mucous membranes) or comorbidities associated with volume depletion (i.e., gastroenteritis) and prerenal azotemia in children with CM or SMA (*p* > 0.05 for all). Among children with severe malaria, prerenal azotemia was associated with an increased heart rate (*p* = 0.004) and lower weight-for-age (*p* = 0.01) and weight-for-height *z* scores (*p* = 0.004). Children with severe malaria and prerenal azotemia had a higher parasite density (*p* = 0.001) and parasite biomass (plasma HRP-2, *p* = 0.002) than children without prerenal azotemia. In a multivariable model, a one-unit increase in the natural log of plasma HRP2 was associated with an aOR of prerenal injury of 1.28 (1.15, 1.66) following adjustment for age, sex, heart rate, and weight-for-age and weight-for-height *z* score.

In bivariate analysis, AKI was associated with disease severity, hemoglobinuria, low glucose concentrations, high concentrations of lactate, total bilirubin, lactate dehydrogenase (LDH), and parasite biomass (plasma HRP-2) (Table 3, Additional file 1: Table S3-S7). However, in multivariable models, LDH was the only measure independently associated with AKI in both CM and SMA following adjustment for multiple comparisons (Additional file 1: Table S4-S5). A one-unit increase in the natural log of LDH was associated with adjusted odds ratios (aOR) of AKI of 5.10 (95% CI 2.22, 11.69) and 8.62 (3.02, 24.60) in CM and SMA, respectively. History of nephrotoxic medication use was not associated with AKI at admission.

**Table 2:** Supportive care and treatment in children with severe malaria according to AKI status

	Severe malarial anemia (*n* = 219)			Cerebral malaria (*n* = 260)		
	No AKI (*n* = 164)	AKI (*n* = 55)	*p* value	No AKI (*n* = 147)	AKI (*n* = 113)	*p* value
Supportive care						
Oxygen	22 (6.7)	7 (12.7)	0.160	71 (48.3)	76 (67.3)	0.002
Antipyretics	161 (98.2)	53 (96.4)	0.437	138 (93.9)	104 (92.0)	0.562
Medications						
Quinine	127 (77.4)	44 (80.0)	0.691	131 (89.1)	99 (87.6)	0.707
Artesunate/artemether	55 (33.5)	16 (29.1)	0.619	43 (29.3)	39 (34.5)	0.420
Anticonvulsants	1 (0.6)	2 (3.6)	0.156	109 (74.2)	96 (85.0)	0.034
Furosemide	32 (19.5)	11 (20.0)	0.937	9 (6.1)	26 (23.0)	< 0.0001
Bolus dextrose	28 (17.1)	28 (50.9)	< 0.0001	132 (89.8)	91 (80.5)	0.034
Intravenous fluids	12 (7.3)	5 (9.1)	0.671	24 (16.3)	32 (28.3)	0.020
0.9% saline	10 (6.1)	4 (7.3)	0.754	18 (12.2)	24 (21.2)	0.051
Ringer’s lactate	0 (0.0)	0 (0.0)	–	2 (1.4)	4 (3.5)	0.246
10% dextrose	0 (0.0)	0 (0.0)	–	4 (2.7)	2 (1.8)	0.700
Bicarbonate	0 (0.0)	0 (0.0)	–	0 (0.0)	1 (0.9)	0.435
Darrow’s	0 (0.0)	1 (1.8)	0.251	1 (0.7)	0 (0.0)	1.000
Albumin	2 (1.2)	0 (0.0)	1.000	2 (1.4)	4 (3.5)	0.408
Blood transfusion	164 (100.0)	55 (100.0)	–	68 (46.3)	81 (71.7)	< 0.0001

Data presented as *n* (%) and compared using Pearson’s chi-square or Fisher’s exact, as appropriate

Children in coma underwent an eye exam using indirect ophthalmoscopy. AKI was associated with an increase in the frequency of peripheral and macular whitening, the frequency and severity of retinal hemorrhages, and the number of retinopathy categories positive (Additional file 1: Table S8-S9). Following adjustment for retinopathy risk factors [20] and multiple comparisons, AKI was associated with peripheral and macular whitening and the number of retinopathy categories positive with aOR 2.60 (95% CI 1.20, 5.21), 2.82 (1.49, 5.30), and 2.30 (1.36, 3.89), respectively. AKI was not significantly associated with vessel color changes or papilloedema (*p* > 0.05). To explore whether AKI was associated with changes in blood-brain barrier integrity, we evaluated albumin concentrations in the CSF of children with CM who had a lumbar puncture to rule out meningitis. A one-unit increase in the natural log of cerebrospinal fluid albumin was associated with an aOR of 2.78 (95% CI 1.56, 4.93; *p* < 0.001) for having AKI on admission.

## Discussion

Acute kidney injury (AKI) is a well-established complication of severe malaria in adults [13], but only recently it has been recognized as a common complication in children with severe malaria [10]. Prior studies established an association between AKI and mortality in severe malaria [9–13]. The present study confirms a strong association of AKI with mortality in children, with over 60% of deaths occurring in children with AKI. This study also demonstrated an association between AKI and persistent neurologic deficits in children with CM, and long-term neurocognitive impairment in children with CM and SMA. Further, this study suggests that AKI in children with severe malaria is associated with CKD. Together, the results show that AKI is a common complication of severe malaria in African children associated with major adverse long-term health outcomes.

The etiology of AKI in pediatric severe malaria is generally attributed to reduced renal perfusion, and this study supports prerenal azotemia as a presenting etiology of AKI. Prerenal azotemia was not associated with clinical measures of dehydration in this cohort apart from an elevated heart rate. However, children with prerenal azotemia had higher parasite density and biomass, consistent with impaired tissue perfusion related to microvascular sequestration of parasitized erythrocytes. Fluid management was conservative with the majority of children receiving intravenous quinine with blood transfusion and maintenance fluids in the first 24 h of hospitalization (Table 2). The relationship between AKI and mortality in the present study does not appear to be related to aggressive fluid replacement therapy. Questions remain regarding the most appropriate and safe approach to fluid resuscitation in the context of severe malaria complicated by severe anemia and impaired tissue perfusion [21, 22], particularly in settings of severe resource constraints where nursing care is limited and mechanical ventilation is unavailable.

**Table 3:** Measures associated with acute kidney injury

	Severe malarial anemia (*n* = 219)			Cerebral malaria (*n* = 260)		
	No AKI (*n* = 164)	AKI (*n* = 55)	*p* value	No AKI (*n* = 147)	AKI (*n* = 113)	*p* value
Demographics						
Age, years	2.8 (2.1, 4.2)	3.2 (2.0, 4.9)	0.375	3.6 (2.7, 5.4)	3.2 (2.2, 4.5)	0.014
Sex, F %	65 (39.6)	21 (38.2)	0.849	59 (40.1)	48 (42.3)	0.705
Weight-for-age *z* score	− 1.5 (− 2.2, − 0.7)	− 1.8 (− 2.4, − 0.6)	0.439	− 1.0 (− 1.8, − 0.4)	− 1.4 (− 1.9, − 0.8)	0.044
Height-for-age *z* score	− 1.0 (− 1.8, − 0.3)	− 1.4 (− 2.6, − 0.5)	0.119	− 0.7 (− 1.3, 0.3)	− 1.0 (− 1.9, − 0.1)	0.024
Weight-for-height *z* score	− 0.9 (− 1.8, − 0.1)	− 0.9 (− 1.6, − 0.1)	0.697	− 1.0 (− 1.8, − 0.1)	− 1.0 (− 1.7, − 0.3)	0.914
HIV-infected, *n* (%)	5 (3.1)	1 (1.9)	1.000	1 (0.7)	4 (4.0)	0.165
Sickle cell disease (HbSS), *n* (%)	17 (10.4)	4 (7.3)	0.605	1 (0.7)	0 (0.0)	0.380
Admission characteristics						
Symptoms						
History of fever, days	4 (3, 5)	3 (2, 5)	0.078	3 (2, 4)	3 (2, 4)	0.537
Tea-colored urine, *n* (%)	23 (14.0)	15 (27.3)	0.025	18 (12.2)	27 (23.9)	0.014
Diarrhea, *n* (%)	10 (6.1)	6 (10.9)	0.235	6 (4.1)	6 (5.3)	0.640
Vomiting, *n* (%)	74 (45.1)	38 (69.1)	0.002	60 (40.8)	38 (33.6)	0.236
Clinical signs						
Temperature, °C	37.7 (37.0, 38.5)	37.7 (36.7, 38.5)	0.976	38.0 (36.9, 38.5)	37.6 (37.0, 38.5)	0.161
Pulse, beats/min	150 (137, 162)	154 (138, 168)	0.158	148 (128, 165)	150 (135, 168)	0.278
Respiratory rate, breaths/min	44 (36, 55)	40 (35, 52)	0.358	44 (34, 54)	44 (36, 56)	0.357
Systolic blood pressure, mmHg	90 (82, 100)	90 (85, 100)	0.708	96 (90, 105)	96 (85, 104)	0.409
Blantyre coma score	5 (5, 5)	5 (5, 5)	–	2 (1, 2)	2 (1, 2)	0.684
Glasgow coma score	15 (15, 15)	15 (15, 15)	–	7 (6, 8)	7 (6, 8)	0.760
Severe dehydration, *n* (%)^1^	4 (2.4)	3 (5.5)	0.371	4 (2.7)	3 (2.7)	1.000
Urine hemoglobin positive, *n* (%)	10 (7.7)	8 (19.5)	0.032	14 (10.8)	28 (33.7)	< 0.0001
Hemoglobinuria, *n* (%)	16 (9.8)	13 (23.6)	0.009	10 (6.9)	21 (18.6)	0.004
Laboratory tests						
Hemoglobin, g/dL	4.0 (3.1, 4.6)	3.8 (3.4, 4.3)	0.942	7.2 (5.7, 9.0)	6.0 (4.8, 7.9)	0.0002
Glucose, mmol/L	6.8 (5.0, 8.2)	5.3 (4.1, 7.2)	0.002	6.9 (5.4, 9.6)	6.1 (4.6, 4.6)	0.032
Lactate, mmol/L	4.6 (2.8, 7.7)	5.5 (3.6, 9.5)	0.052	3.3 (2.0, 6.1)	4.3 (2.8, 8.0)	0.003
WBC, × 10^3^/μL	11.2 (8.2, 15.7)	12.3 (9.2, 22.3)	0.117	8.3 (6.3, 12.3)	10.8 (7.9, 17.4)	0.0008
Platelet, × 10^3^/μL	155 (95, 242)	133 (82, 197)	0.179	62 (35, 112)	51 (33, 109)	0.225
Total bilirubin, mg/dL	1.1 (0.6, 1.9)	1.5 (0.9, 2.9)	0.007	1.4 (0.8, 2.0)	2.0 (1.0, 3.6)	0.0001
Lactate dehydrogenase (LDH), U/L	728 (591, 879)	990 (754, 1510)	< 0.00001	712 (537, 906)	1090 (797, 1513)	< 0.00001
Plasma albumin, g/dL	2.6 (2.4, 3.0)	2.6 (2.3, 3.2)	0.737	2.6 (2.3, 3.0)	2.7 (2.4, 2.9)	0.467
Sodium, mmol/L	134 (132, 136)	135 (132, 137)	0.383	132 (128, 135)	133 (129, 136)	0.180
Peripheral parasite density, parasites/uL	34,680 (10,435, 133,980)	40,070 (6085, 138,470)	0.868	50,820 (13,910, 287,835)	42,140 (8000, 249,560)	0.338
Plasma PfHRP2, ng/mL	886 (326, 2344)	1415 (382, 3453)	0.151	2244 (655, 3965)	4394 (2208, 7469)	< 0.00001
Creatinine, mg/dL	0.31 (0.26, 0.38)	0.53 (0.48, 0.67)	< 0.00001	0.33 (0.29, 0.39)	0.58 (0.49, 0.78)	< 0.00001
BUN, mg/dL	11 (8, 16)	22 (15, 30)	< 0.00001	13 (10, 17)	26 (18, 41)	< 0.00001
Composite disease severity score						
Number of severity criteria^2^	3 (2, 3)	3 (2, 4)	0.0029	4 (3, 4)	4 (3, 5)	0.0004
Nephrotoxic medication history^3^						
NSAIDs^4^	12 (7.3)	3 (5.5)	0.224	15 (10.2)	15 (13.3)	0.442
Gentamicin	2 (1.2)	1 (1.8)	1.000	5 (3.4)	5 (4.4)	0.671
Any nephrotoxic medication^5^	14 (8.5)	4 (7.3)	1.000	19 (12.9)	19 (16.8)	0.379
Number of nephrotoxic medications^6^						
0	148 (91.4)	50 (92.6)	0.835	127 (87.6)	98 (82.9)	0.569
1	13 (8.0)	4 (7.4)		17 (11.7)	18 (16.2)	
2	1 (0.6)	0 (0.0)		1 (0.7)	1 (0.9)	

Continuous measures presented as median (interquartile range) unless otherwise indicated. Continuous measures compared using the Wilcoxon rank-sum test. Count measures compared using the Pearson’s chi-square or Fisher’s exact, as appropriate

^1^Severe dehydration (*n* = 14) was identified by the presence of sunken eyes (*n* = 12) or decreased skin turgor (*n* = 4), hemoglobinuria defined as tea-colored urine on microscopy without red blood cells

^2^Number of WHO criteria for severe malaria present (description in Additional file 1: Table S1)

^3^AKI was retrospectively assessed on stored blood samples, and data on AKI was not available on admission

^4^Non-steroidal anti-inflammatory drug (NSAID), included ibuprofen (*n* = 5), acetylsalicylic acid (*n* = 5), and diclophenac (*n* = 35)

^5^NSAID or gentamicin

^6^Sum of ibuprofen, acetylsalicylic acid, diclophenac, and gentamicin

AKI was associated with the presence, severity, and extent of retinopathy and the presence of coma and blood-brain barrier impairment in this study. We hypothesize microvascular changes in the retina and brain are associated with generalized systemic microvascular changes in severe malaria. Parasite biomass was related to prerenal injury, consistent with studies in adults with severe malaria-associated AKI [23]. Ultrastructural examination of kidney tissue of adults with fatal malaria reveals parasite sequestration in glomerular and tubulointerstitial vessels and monocyte accumulation in glomerular capillaries [24]. In children, renal sequestration is less frequent, but still occurs, and may potentially contribute to AKI [8].

Histologic findings of malaria pigment in distal convoluted tubules [8] are consistent with the reports of intravascular hemolysis and increased cell-free hemoglobin and heme in malaria-associated AKI [25, 26]. In the present study, LDH was strongly associated with AKI, supporting the hypothesis that hemolysis contributes to oxidative stress and pigment nephropathy. This is further supported by increased bilirubin, another marker of hemolysis, and increased frequency of hemoglobinuria in AKI. A recent clinical trial showed a renoprotective effect of acetaminophen in adults with severe malaria and intravascular hemolysis [27]. Retrospective data from pediatric cardiac surgery suggest early postoperative acetaminophen reduces hemoglobin-mediated oxidative stress and AKI [28]. The present study supports a role for hemolysis and increased cell-free hemoglobin in pediatric AKI and highlights the need for renoprotective adjunctive therapies to reduce the incidence and severity of AKI in pediatric malaria.

In this study, AKI was associated with CKD at 1-year follow-up, consistent with a growing body of evidence that AKI is a risk factor for short-term [29] and long-term CKD and end-stage kidney disease in children [30]. Although AKI can result in persistent and progressive renal dysfunction, even complete recovery is associated with subsequent risk of developing CKD [15]. For example, between 8 and 61% of children who recovered baseline renal function following hemolytic uremic syndrome developed renal complications within 5 to 10 years [31]. The present study is the first to report an association between AKI and CKD in the context of severe malaria. We are currently planning to follow up the study participants to re-evaluate kidney function 5 to 12 years after severe malaria.

Distant organ injury in AKI is well-described, and neurologic complications including central nervous system dysfunction, decreased mental status, and seizures are associated with AKI [32]. The presence of mild-to-moderate CKD is associated with deficits in academic achievement, executive function, and visual and verbal memory [33], but to our knowledge, this is the first report of an association between AKI and neurocognitive impairment. Well-recognized risk factors for neurocognitive impairment in severe malaria relate to neurological complications on admission and inflammatory and neuroactive metabolites in cerebrospinal fluid [2–5, 34–36]. Increased cerebrospinal fluid albumin in children with CM and AKI suggests blood-brain barrier impairment. Many processes known to be important in cognitive impairment, including endothelial dysfunction, oxidative stress, and inflammation, also relate to AKI [32]. The relationship of AKI to neurocognitive impairment could reflect the contribution of these processes to both clinical entities, or reflect an independent role for AKI in neurocognitive impairment, through mechanisms still to be defined.

In the present study, the frequency of CKD was higher in children treated with artemisinin derivatives, though with the small number of children with CKD, these differences did not approach statistical significance. This study highlights the need for additional studies to assess CKD in children treated with artemisinin derivatives. The association between AKI and mortality and neurocognitive impairment was seen in children treated with either drug, so this association occurs independently of severe malaria treatment.

Strengths of the study include its sample size, rigorous clinical definitions, prospective design, and careful neurocognitive follow-up. Including the community controls to estimate premorbid creatinine likely increased our ability to detect AKI. Further, our findings are consistent irrespective of the method used to define premorbid creatinine, suggesting an epidemiologic approach is valid when premorbid creatinine measurements or community controls are unavailable. The study is the first to assess the relationship between AKI and CKD in malaria, and the largest pediatric study to date to assess post-AKI CKD.

Among the study limitations are the retrospective definition of AKI, using a single measurement of creatinine on admission and estimating pre-illness creatinine from community control data. The study definition likely underestimates AKI [37]. Further investigation, including detailed urinalysis and renal biopsy findings, is also needed to understand the pathophysiology of AKI in children with severe malaria. Additional studies are needed to evaluate AKI in low-resource settings to understand whether the findings in this paper are generalizable to all critically ill hospitalized children or specific to severe malaria-associated AKI.

## Conclusions

In summary, the present study supports a growing body of evidence that AKI is a risk factor for long-term morbidity and mortality in malaria, providing new evidence that AKI is a risk factor for sustained neurocognitive impairment in survivors. Further, this study suggests that children with severe malaria-associated AKI may be at risk of developing CKD. This has far-reaching implications for the burden of non-communicable disease in Uganda, where the prevalence of CKD is estimated at 18.1% [38] and renal replacement therapy is not available in most areas. To make progress towards the International Society of Nephrology initiative to eliminate preventable deaths from AKI worldwide by 2025 (0by25) [39], a concerted effort to improve diagnosis, and management, of AKI in resource-constrained malaria-endemic settings is urgently needed.

## Additional file

Additional file 1: I. Methods. Supplementary methods describing the study population and additional details on assessment of disease severity, kidney function, retinopathy, neurocognitive evaluation, and statistical analysis. II. Results. Relationship between AKI and nephrotoxic medication use during hospitalization. III. Supplementary tables. Table S1-S13. (DOCX 89 kb)

## Abbreviations

AKI: Acute kidney injury
CC: Community children
CI: Confidence interval
CKD: Chronic kidney disease
CM: Cerebral malaria
eGFR: Estimated glomerular filtration rate
HOME: Home Observation for the Measurement of the Environment
HR: Hazards ratio
LDH: Lactate dehydrogenase
OR: Odds ratio
SMA: Severe malarial anemia
