# Supplementary material for: Acute kidney injury is associated with impaired cognition and chronic kidney disease in a prospective cohort of children with severe malaria
Source: BMC Med. 2019 May 21;17:98. doi: 10.1186/s12916-019-1332-7 (PMC6528242; doi:10.1186/s12916-019-1332-7)
Supplement: Supplementary file 1 — I. Methods. Supplementary methods describing the study population and additional details on assessment of disease severity, kidney function, retinopathy, neurocognitive evaluation, and statistical analysis. II. Results. Relationship between AKI and nephrotoxic medication use during hospitalization. III. Supplementary tables. Table S1-S13. (DOCX 89 kb) [file 12916_2019_1332_MOESM1_ESM.docx]

**Additional File 1**

Supplement to: Conroy AL, Opoka RO, Bangirana, P, et al. Acute kidney injury is associated with impaired cognition and chronic kidney disease in a prospective cohort of children with severe malaria

[I. Methods 3](#_Toc1579515)

[1) Study Population 3](#_Toc1579516)

[2) Assessment of disease severity 3](#_Toc1579517)

[3) Assessment of kidney function 4](#_Toc1579518)

[4) Ophthalmologic assessment of retinopathy 5](#_Toc1579519)

[5) Neurocognitive Assessment 5](#_Toc1579520)

[7) Statistical Analysis 6](#_Toc1579521)

[II. Supplementary Results 7](#_Toc1579522)

[Relationship between AKI and nephrotoxic medication use 7](#_Toc1579523)

[III. Supplementary Tables 8](#_Toc1579524)

[Table S1. Severe malaria criteria used to create composite disease severity score 8](#_Toc1579525)

[Table S2. Comparing AKI according to the method used to estimate baseline creatinine 9](#_Toc1579526)

[Table S3. Measures associated with AKI using an epidemiologic approach to define AKI 10](#_Toc1579527)

[Table S4. Logistic regressions evaluating independent predictors of AKI in children with CM using community controls to define AKI 12](#_Toc1579528)

[Table S5. Logistic regressions evaluating independent predictors of AKI in children with SMA using community controls to define AKI 12](#_Toc1579529)

[Table S6. Logistic regressions evaluating independent predictors of AKI in children with CM using an epidemiologic approach to define AKI 13](#_Toc1579530)

[Table S7. Logistic regressions evaluating independent predictors of AKI in children with SMA using an epidemiologic approach to define AKI 13](#_Toc1579531)

[Table S8. AKI and retinopathy using community controls to define AKI 14](#_Toc1579532)

[Table S9. AKI and retinopathy using an epidemiologic approach to define AKI 14](#_Toc1579533)

[Table S10. Association between AKI, clinical recovery, and survival using community controls to define AKI 15](#_Toc1579534)

[Table S11. Association between AKI, clinical recovery, and survival using an epidemiologic approach to define AKI 15](#_Toc1579535)

[Table S12. Odds ratios of having neurocognitive impairment in children with AKI on hospital admission using community controls to define AKI 16](#_Toc1579536)

[Table S13. Odds ratios of having neurocognitive impairment in children with AKI on hospital admission using an epidemiologic approach to define AKI 16](#_Toc1579537)

# Methods

## Study Population

All children with severe malaria had *P. falciparum* on blood smear. Children with cerebral malaria (CM) had a coma with no other identifiable cause ruling out meningitis, a prolonged postictal state, or hypoglycemia-associated coma reversed by a glucose infusion. Children with severe malarial anemia (SMA) had hemoglobin level ≤5g/dL. Exclusion criteria included known chronic illness requiring medical care, known developmental delay, or history of coma, head trauma, hospitalization for malnutrition, or cerebral palsy. Additional exclusion criteria in children with SMA included impaired consciousness on physical examination, other clinical evidence of central nervous system disease, or more than one seizure before admission. Additional exclusion criteria for the community controls (CC) included any illness requiring medical care within the previous four weeks or major medical or neurologic abnormalities at screening physical examination.

Children were managed according to the Ugandan national treatment guidelines at the time of the study, which included intravenous quinine treatment followed by oral quinine for severe malaria while admitted, and artemisinin combination therapy for outpatient follow-up therapy. All children with severe anemia received a blood transfusion.

## Assessment of disease severity

To adjust for the severity of acute disease in assessing long-term outcomes, we generated a composite disease severity score based on the number of World Health Organization criteria for severe malaria present including: clinical manifestations of severe malaria (impaired consciousness, respiratory distress, multiple convulsions, prostration, shock, abnormal bleeding, and jaundice); and laboratory indices of disease severity (severe anemia, hypoglycemia, hyperlactatemia, hyperparasitemia)^1^. Thus, each child with severe malaria could have a score ranging from 1 to 11. If a data item were missing for a child, it was assumed normal. Renal impairment was excluded from the disease severity score as it was included in all models as the predictor of interest.

## Assessment of kidney function

To estimate pre-morbid creatinine levels, we used two approaches. The first approach used linear regression and enrollment creatinine and height data from the community controls to construct a creatinine-for-height curve to predict baseline creatinine for the children admitted with severe malaria based on their admission height. One CC child with evidence of kidney dysfunction was excluded from the model, and improved the model fit (adjusted R^2^) from 14% to 22%. The second approach assumed a normal estimated glomerular filtration rate (eGFR) of 120mL/min/1.73m^2^ and used the Bedside Schwartz equation^2^ to back calculate baseline creatinine, as previously described^3^.

As the Bedside Schwartz equation estimates renal function in children using the child’s height, we conducted a comprehensive review of heights recorded over the study period to identify outliers. Individuals' height outliers were identified using linear mixed effects models with age and sex as fixed effects and patient specific random intercepts and slopes^4^. This approach allowed us to use the longitudinal height data collected over two years to flag heights with a standardized residual < –2 or > 2 for further review. Twenty-eight outliers were identified and adjusted using growth data from follow-up. Height at admission was missing for 5% of children (15 study deaths, 7 surviving children with follow-up). For survivors with height data at follow-up, the follow-up data were used to estimate admission height. In non-survivors, WHO population height-for-age growth curves were used to impute a height, assuming the child was on the 10^th^ percentile of height for age and sex, corresponding to the growth curve of the population.

Hemoglobinuria was assessed three ways: parental report of “tea-coloured” urine on admission, a dipstick test for urine hemoglobin, and examination of the urine by microscopy to identify tea-coloured urine without red blood cells present. All three ways to assess hemoglobinuria were associated with AKI. As hemoglobinuria assessed by microscopy showed the strongest relationship with AKI, and was assessed by trained laboratory personnel, we used this objective measure in the analysis.

## Ophthalmologic assessment of retinopathy

Children were assessed for retinopathy using indirect ophthalmoscopy on admission. Pupils were dilated with sequential instillation of cyclopentolate 1% and tropicamide 1% and an eye exam was conducted using a binocular indirect ophthalmoscope 30–60 minutes later. Retinal exams were conducted by study medical officers. Initial training of study investigators, a Ugandan ophthalmologist, and study medical officers was conducted by an ophthalmologist experienced in evaluating malaria retinopathy.

## Neurocognitive Assessment

Neurologic status was assessed by a study clinician at discharge and at one and two years follow-up. Neurologic deficits were assessed through a standardized neurologic exam conducted by a study physician. Medical examiners and neuropsychology testers were blinded to group. A neurologic deficit was defined as speech difficulties including aphasia or speech regression, visual impairment or blindness, motor deficits (hypertonia, hypotonia, spasticity, cranial nerve palsy), movement disorders (tremor, dystonia, choreoathetoid), ataxia, hearing deficits, hyporeflexia, or Babinski sign. Cognitive testing was conducted by trained neuropsychological testers without knowledge of the child’s disease history. Neurocognitive evaluations were conducted by assessors with demonstrated proficiency in testing. The KABC-II has been validated for use in Ugandan children^5,6^. The Mullen has been used in Ugandan children to investigate the effect of malaria on cognition and to evaluate interventions to enhance cognition in HIV infected children^7,8^. An assessment of enrichment in the home environment and guardian interaction with the child was conducted by a trained home visitor.

1. **Health assessment at follow-up appointments**

At one and two years’ follow-up, children were screened by the medical team to rule out active illness, as that could affect neurocognitive evaluation. Children underwent a follow-up history and physical exam and were rescheduled if they had fever, vomiting, severe diarrhea, difficulty breathing, severe cough or active convulsions. In the event of active illness, they were sent to the hospital ward for evaluation and rescheduled for the follow-up appointment when symptom-free. An assessment of prior illness requiring treatment or hospitalization was also assessed at each visit, along with a list of medications taken. For all children meeting a definition of CKD, a chart review was conducted to ensure there were no active illnesses or recent medications that may have led to an episode of AKI. One child had a cough without fever on the study visit and was considered well enough to proceed with the study visit. The child had not taken any medications prior to the visit.

## Statistical Analysis

Age-adjusted z scores for cognitive outcomes were created using the scores of the CC. For each outcome, the z score was computed as (actual score − mean score for child’s age)/standard deviation (SD), where the mean score for age and SD were computed by fitting a mixed linear model to data from all available visits for CC (allowing correlated errors for a child’s multiple visits). By construction, z scores have mean 0 and SD 1 in the reference population (CC) over all time points. In investigating the relationship between AKI and neurocognitive outcomes, we considered a number of potential characteristics related to disease severity and complications that have been implicated in adverse neurocognitive outcomes. As this study included children with CM and SMA, we did not include data related to coma duration as it was not relevant to all children. However, we adjusted for the presence of coma in analyses. We considered hypoglycemia, multiple seizures before or during admission, and hyperpyrexia, but only the number of seizures during admission was included because it was related to neurocognitive outcomes and improved model fit. For continuous measures the odds ratio corresponds to a one-unit increase unless a transformation is otherwise indicated.

# Supplementary Results

## Relationship between AKI and nephrotoxic medication use

As AKI was retrospectively defined and information on AKI status was not available when clinical decisions were made in-hospital, we evaluated the relationship between AKI and use of nephrotoxic medications. Medication history was reviewed and data were extracted on the use of aminoglycoside antibiotics (Gentamicin), and non-steroidal anti-inflammatory drug (NSAID) use (ibuprofen, acetylsalicylic acid, diclophenac). Children were classified as having received a nephrotoxic medication if they received Gentamicin or a NSAID, and the number of nephrotoxic medications received prior to admission. Data are presented in Table 2 and Table S3 stratified by severe malaria group (cerebral malaria, or severe malarial anemia). Although use of acetylsalicylic acid was infrequent overall at 0.8% and was not associated with AKI on admission, it was associated with increased mortality with 40.0% of children receiving acetylsalicylic acid dying in hospital compared to 6.5% of children who did not receive acetylsalicylic acid (p=0.003).

We further evaluated whether long-term renal recovery was related to use of nephrotoxic drug use during hospitalization based on whether children were administered Gentamicin. Gentamicin was prescribed to 17 children (n=7, CM; n=10, SMA), and there was no difference in prescription based on AKI status on admission (p>0.05 for both children with CM and SMA). None of the 17 children administered Gentamicin in hospital developed CKD or died during the study.

# Supplementary Tables

## Table S1. Severe malaria criteria used to create composite disease severity score

| **Criteria** | **N (%) missing** | **Frequency** |
| --- | --- | --- |
| **Clinical Manifestations** |  |  |
| Impaired consciousness, n (%) | 0 (0) | 257 (53.7) |
| Respiratory distress, n (%) | 0 (0) | 37 (7.7) |
| Multiple Convulsions, n (%) | 0 (0) | 161 (33.6) |
| Prostration, n (%) | 0 (0) | 361 (75.4) |
| Abnormal Bleeding, n (%) | 0 (0) | 9 (1.9) |
| Shock, n (%) | 0 (0) | 1 (0.2) |
| Jaundice, n (%) | 0 (0) | 287 (59.9) |
| **Laboratory Indices** |  |  |
| Severe anemia, n (%) | 0 (0) | 276 (57.6) |
| Hypoglycemia, n (%) | 12 (2.5) | 27 (5.8) |
| Hyperlactatemia, n (%) | 35 (7.3) | 183 (41.2) |
| Hyperparasitemia, n (%) | 2 (0.4) | 52 (10.9) |

Severe anemia (Hb<5g/dL), hypoglycemia (glucose <2.2mmol/L), hyperlactatemia (lactate >5.5mmol/L), hyperparasitemia (parasite density >500,000/uL or 10%).

Renal dysfunction/ acute kidney injury was excluded from the disease severity score.

In constructing the disease severity score, it was assumed a complication was absent if data were missing.

## Table S2. Comparing AKI according to the method used to estimate baseline creatinine

|  | Population specific approach to estimate baseline creatinine using community controls | | | |  |
| --- | --- | --- | --- | --- | --- |
| Epidemiologic approach to estimate baseline creatinine^3^ | No AKI | Stage 1 AKI | Stage 2 AKI | Stage 3 AKI | Total |
| No AKI | 311 | **39** | 0 | 0 | 350 |
| Stage 1 AKI | 0 | 57 | **13** | 0 | 70 |
| Stage 2 AKI | 0 | 0 | 36 | **4** | 40 |
| Stage 3 AKI | 0 | 0 | 0 | 19 | 19 |
| Total | 311 | 96 | 49 | 23 | 479 |

Assumed eGFR 120mL/min/1.73m^2^ to estimate baseline creatinine

## Table S3. Measures associated with AKI using an epidemiologic approach to define AKI

|  | **Severe Malarial Anemia (n=219)** | | | **Cerebral Malaria (n=260)** | | |
| --- | --- | --- | --- | --- | --- | --- |
|  | **No AKI (n=179)** | **AKI (n=40)** | **P-value** | **No AKI (n=171)** | **AKI (n=89)** | **P-value** |
| **Demographics** |  |  |  |  |  |  |
| Age, years | 2.8 (2.1, 4.4) | 3.3 (2.0, 4.7) | 0.530 | 3.6 (2.7, 5.2) | 3.2 (2.2, 4.4) | 0.022 |
| Sex, F % | 70 (39.1) | 16 (40.0) | 0.917 | 69 (40.4) | 38 (42.7) | 0.715 |
| Weight-for-age z score | -1.5 (-2.2, -0.7) | -1.5 (-2.3, -0.6) | 0.795 | -1.0 (-1.9, -0.4) | -1.4 (-1.9, -0.9) | 0.087 |
| Height-for-age z score | -1.0 (-1.9, -0.3) | -1.4 (-2.5, -0.5) | 0.136 | -0.8 (-1.3, 0.2) | -1.0 (-2.0, -0.2) | 0.042 |
| Weight-for-height z score | -1.0 (-1.8, -0.1) | -0.8 (-1.6, 0.1) | 0.213 | -0.9 (-1.8, -0.2) | -1.1 (-1.7, -0.3) | 0.987 |
| HIV-infected, n (%) | 5 (2.8) | 1 (2.6) | 1.000 | 2 (1.2) | 3 (3.9) | 0.333 |
| Sickle cell disease (HbSS), n (%) | 18 (10.1) | 3 (7.5) | 0.773 | 1 (0.6) | 0 (0.0) | 1.000 |
| **Admission characteristics** |  |  |  |  |  |  |
| **Symptoms** |  |  |  |  |  |  |
| History of fever, days | 4 (3, 6) | 3 (2, 4) | 0.057 | 3 (2, 4) | 3 (3, 4) | 0.923 |
| Tea coloured urine, n (%) | 26 (14.5) | 12 (40.0) | 0.019 | 23 (13.5) | 22 (24.7) | 0.023 |
| Diarrhea, n (%) | 10 (5.6) | 6 (15.0) | 0.039 | 7 (4.1) | 5 (5.6) | 0.578 |
| Vomiting, n (%) | 83 (46.4) | 29 (72.5) | 0.003 | 68 (39.8) | 30 (33.7) | 0.339 |
| **Clinical signs** |  |  |  |  |  |  |
| Temperature, ºC | 37.7 (36.9, 38.5) | 37.8 (36.9, 38.4) | 0.955 | 37.9 (37.1, 38.5) | 37.6 (37.0, 38.5) | 0.208 |
| Pulse, beats/minute | 150 (137, 162) | 152 (137, 166) | 0.462 | 148 (128, 164) | 152 (135, 172) | 0.186 |
| Respiratory rate, breaths/minute | 44 (36, 54) | 44 (36, 54) | 0.889 | 44 (34, 56) | 44 (36, 56) | 0.752 |
| Systolic blood pressure, mmHg | 90 (82, 100) | 90 (85, 99) | 0.847 | 96 (87, 105) | 96 (88, 104) | 0.705 |
| Blantyre coma score | 5 (5, 5) | 5 (5, 5) | ----- | 2 (1, 2) | 2 (1, 2) | 0.634 |
| Glasgow coma score | 15 (15, 15) | 15 (15, 15) | ------ | 7 (7, 8) | 7 (6, 8) | 0.651 |
| Severe dehydration, n (%) ^1^ | 5 (2.8) | 2 (5.0) | 0.614 | 4 (2.3) | 3 (3.4) | 0.694 |
| Urine hemoglobin positive, n (%) | 13 (9.2) | 5 (17.2) | 0.196 | 19 (12.5) | 23 (37.7) | <0.0001 |
| Hemoglobinuria, n (%) | 18 (10.1) | 11 (27.5) | 0.003 | 13 (7.7) | 18 (20.2) | 0.003 |
| **Laboratory tests** |  |  |  |  |  |  |
| Hemoglobin, g/dL | 3.9 (3.2, 4.5) | 3.8 (3.2, 4.3) | 0.643 | 7.1 (5.6, 8.9) | 6.0 (4.8, 7.7) | 0.002 |
| Glucose, mmol/L | 6.8 (5.0, 8.2) | 4.7 (3.5, 5.7) | 0.0006 | 6.7 (5.5, 9.4) | 6.1 (4.5, 8.6) | 0.037 |
| Lactate, mmol/L | 4.7 (2.8, 7.6) | 5.5 (3.7, 10.8) | 0.031 | 3.4 (2.0, 6.0) | 4.5 (3.0, 8.5) | 0.0007 |
| WBC, x10^3^/μL | 11.2 (8.2, 15.8) | 12.5 (9.4, 25.9) | 0.138 | 8.7 (6.3, 12.5) | 10.9 (8.1, 19.5) | 0.0005 |
| Platelet, x10^3^/μL | 157 (96, 241) | 127 (76, 193) | 0.074 | 61 (34, 112) | 56 (35, 109) | 0.740 |
| Total bilirubin, mg/dL | 1.1 (0.6, 1.9) | 1.6 (1.3, 3.8) | 0.0002 | 1.4 (0.8, 2.1) | 2.1 (1.1, 3.7) | 0.0001 |
| Lactate dehydrogenase, U/L | 730 (596, 900) | 1159 (785, 1775) | <0.00001 | 741 (555, 938) | 1126 (831, 1698) | <0.00001 |
| Plasma Albumin, g/dL | 2.6 (2.4, 3.0) | 2.7 (2.3, 3.2) | 0.469 | 2.6 (2.3, 2.9) | 2.7 (2.4, 3.0) | 0.333 |
| Sodium, mmol/L | 134 (132, 136) | 134 (131, 137) | 0.788 | 132 (128, 135) | 133 (129, 136) | 0.418 |
| Peripheral parasite density, parasites/uL | 35180 (10360, 165780) | 31480 (6660, 107820) | 0.715 | 48430 (13370, 292385) | 42140 (6450, 238270) | 0.355 |
| Plasma PfHRP2 concentration, ng/mL | 847 (312, 2438) | 1901 (458, 3705) | 0.018 | 2308 (845, 4202) | 4631 (2262, 7478) | <0.00001 |
| Creatinine, mg/dL | 0.32 (0.27, 0.39) | 0.59 (0.51, 0.71) | <0.00001 | 0.35 (0.29, 0.42) | 0.65 (0.53, 0.90) | <0.00001 |
| BUN, mg/dL | 12 (8, 16) | 25 (18, 40) | <0.00001 | 14 (10, 18) | 30 (21, 46) | <0.00001 |
| **Composite disease severity score** |  |  |  |  |  |  |
| Number of severity criteria^2^ | 3 (2, 3) | 2 (3, 4) | 0.0005 | 4 (3, 5) | 4 (3, 5) | 0.006 |
| **Nephrotoxic medication history** |  |  |  |  |  |  |
| Non-steroidal anti-inflammatory drugs^3^ | 12 (6.7) | 3 (7.5) | 0.741 | 18 (10.5) | 12 (13.5) | 0.479 |
| Gentamicin | 2 (1.1) | 1 (2.5) | 0.456 | 8 (4.7) | 2 (2.3) | 0.333 |
| Any nephrotoxic medication^4^ | 14 (7.8) | 4 (10.0) | 0.749 | 25 (14.6) | 13 (14.6) | 0.998 |
| Number of nephrotoxic medications^5^  0  1  2 | 163 (92.1)  13 (7.3)  1 (0.6) | 35 (89.7)  4 (10.3)  0 (0.0) | 0.747 | 145 (85.8)  23 (13.6)  1 (0.6) | 74 (85.1)  12 (13.8)  1 (1.2) | 0.890 |

Continuous measures presented as median (interquartile range) unless otherwise indicated. Continuous measures compared using Wilcoxon rank sum test.

Count measures compared using Pearson’s Chi-Square or Fisher’s exact, as appropriate.

^1^Dehydration (n=14) was identified by the presence of sunken eyes (n=12) or decreased skin turgor (n=4), hemoglobinuria defined as tea coloured urine on microscopy without red blood cells.

**^2^** Number of WHO criteria for severe malaria present (description in Additional file 1, Table S1)

^3^ Included ibuprofen (n=5), acetylsalicylic acid (n=5), diclophenac (n=35)

^4^ Non-steroidal anti-inflammatory drug or Gentamicin

^5^ Sum of ibuprofen, acetylsalicylic acid, diclophenac, Gentamicin

## Table S4. Logistic regressions evaluating independent predictors of AKI in children with CM using community controls to define AKI

|  | **N** | **Percent missing** | **OR (95% CI)** | **P value** | **aOR (95% CI)** | **P value** |
| --- | --- | --- | --- | --- | --- | --- |
| Age, years | 260 | 0 | 0.87 (0.76, 0.999) | 0.048 | 1.02 (0.85, 1.21) | 0.860 |
| Sex, F | 260 | 0 | 1.10 (0.67, 1.81) | 0.704 | 1.44 (0.76, 2.74) | 0.266 |
| Weight-for-age z score | 260 | 0 | 0.80 (0.63, 1.02) | 0.071 | 0.78 (0.57, 1.08) | 0.134 |
| Height for age z score | 260 | 0 | 0.78 (0.64, 0.94) | 0.010 | 0.80 (0.62, 1.02) | 0.076 |
| Hemoglobinuria | 258 | 0.8 | 3.08 (1.39, 6.85) | 0.006 | 2.29 (0.83, 6.32) | 0.111 |
| Hemoglobin, g/dL | 260 | 0 | 0.82 (0.73, 0.92) | 0.001 | 1.01 (0.87, 1.17) | 0.928 |
| Glucose, mmol/L | 258 | 0.8 | 0.92 (0.85, 0.99) | 0.019 | 0.92 (0.84, 1.01) | 0.085 |
| Lactate, mmol/L | 241 | 7.3 | 1.14 (1.06, 1.24) | 0.001 | 1.03 (0.93, 1.14) | 0.556 |
| WBC, x10^3^/μL | 255 | 1.9 | 1.07 (1.03, 1.11) | <0.0001 | 1.05 (1.00, 1.11) | 0.064 |
| Total bilirubin, mg/dL | 257 | 1.2 | 1.28 (1.09, 1.49) | 0.002 | 1.05 (0.91, 1.21) | 0.487 |
| LDH^1^, u/L | 257 | 1.2 | 9.49 (4.69, 19.23) | <0.0001 | 5.10 (2.22, 11.69) | <0.0001* |
| Plasma HRP2^1^, ng/mL | 260 | 0 | 1.54 (1.25, 1.91) | <0.0001 | 1.27 (0.95, 1.70) | 0.103 |

^1^log(e)transformed

*Significant following Holm’s correction for multiple comparisons (n=12)

## Table S5. Logistic regressions evaluating independent predictors of AKI in children with SMA using community controls to define AKI

|  | **N** | **Percent Missing** | **OR (95% CI)** | **P value** | **aOR (95% CI)** | **P value** |
| --- | --- | --- | --- | --- | --- | --- |
| Age, years | 219 | 0 | 1.07 (0.90, 1.27) | 0.456 | 0.99 (0.78, 1.27) | 0.949 |
| Sex, F | 219 | 0 | 0.94 (0.50, 1.76) | 0.849 | 0.83 (0.37, 1.86) | 0.649 |
| Height for age z score | 219 | 0 | 0.82 (0.66, 1.02) | 0.071 | 0.71 (0.54, 0.94) | 0.018 |
| Hemoglobinuria, n | 219 | 0 | 2.86 (1.28, 6.42) | 0.011 | 1.40 (0.44, 4.45) | 0.570 |
| History of fever, days | 219 | 0 | 0.89 (0.79, 1.03) | 0.111 | --- | ---- |
| Vomiting, n | 219 | 0 | 2.72 (1.42, 5.20) | 0.003 | 2.57 (1.15, 5.76) | 0.022 |
| Glucose, mmol/L | 209 | 4.6 | 0.85 (0.75, 0.97) | 0.014 | 0.88 (0.76, 1.02) | 0.083 |
| Lactate, mmol/L | 203 | 7.3 | 1.08 (1.00, 1.18) | 0.064 | 1.01 (0.91, 1.13) | 0.849 |
| Total bilirubin, mg/dL | 218 | 0.5 | 1.22 (1.04, 1.44) | 0.016 | 1.07 (0.83, 1.39) | 0.593 |
| LDH^1^, U/L | 217 | 0.9 | 8.05 (3.57, 18.14) | <0.0001 | 8.62 (3.02, 24.60) | <0.0001* |

^1^ log(e)transformed

*Significant following Holm’s correction for multiple comparisons (n=8)

## Table S6. Logistic regressions evaluating independent predictors of AKI in children with CM using an epidemiologic approach to define AKI

|  | **N** | **Percent missing** | **OR (95% CI)** | **P value** | **aOR (95% CI)** | **P value** |
| --- | --- | --- | --- | --- | --- | --- |
| Age, years | 260 | 0 | 0.86 (0.75, 1.00) | 0.046 | 0.98 (0.82, 1.18) | 0.843 |
| Sex, F | 260 | 0 | 1.10 (0.66, 1.85) | 0.715 | 1.27 (0.66, 2.46) | 0.476 |
| Height for age z score | 260 | 0 | 0.79 (0.65, 0.97) | 0.025 | 0.82 (0.64, 1.04) | 0.104 |
| Hemoglobinuria, n | 258 | 0.8 | 3.04 (1.41, 6.55) | 0.004 | 2.62 (1.00, 6.87) | 0.051 |
| Hemoglobin, g/dL | 260 | 0 | 0.88 (0.79, 0.98) | 0.027 | 1.09 (0.93, 1.27) | 0.280 |
| Glucose, mmol/L | 258 | 0.8 | 0.92 (0.85, 0.99) | 0.032 | 0.94 (0.85, 1.04) | 0.219 |
| Lactate, mmol/L | 241 | 7.3 | 1.16 (1.07, 1.25) | <0.0001 | 1.04 (0.95, 1.15) | 0.384 |
| WBC, x10^3^/μL | 255 | 1.9 | 1.07 (1.03, 1.11) | <0.0001 | 1.05 (1.00, 1.11) | 0.048 |
| Total bilirubin, mg/dL | 257 | 1.2 | 1.27 (1.10, 1.46) | 0.001 | 1.06 (0.93, 1.20) | 0.421 |
| LDH^1^, u/L | 257 | 1.2 | 8.94 (4.46, 17.91) | <0.0001 | 5.27 (2.37, 11.69) | <0.0001* |
| Plasma HRP2^1^, ng/mL | 260 | 0 | 1.49 (1.19, 1.85) | <0.0001 | 1.22 (0.91, 1.64) | 0.185 |

^1^ log(e)transformed

*Significant following Holm’s correction for multiple comparisons (n=11)

## Table S7. Logistic regressions evaluating independent predictors of AKI in children with SMA using an epidemiologic approach to define AKI

|  | **N** | **Percent Missing** | **OR (95% CI)** | **P value** | **aOR (95% CI)** | **P value** |
| --- | --- | --- | --- | --- | --- | --- |
| Age, years | 219 | 0 | 1.05 (0.87, 1.28) | 0.590 | 0.90 (0.65, 1.25) | 0.535 |
| Sex, F | 219 | 0 | 1.04 (0.52, 2.09) | 0.917 | 1.04 (0.40, 2.73) | 0.933 |
| Hemoglobinuria, n | 219 | 0 | 3.39 (1.45, 7.92) | 0.005 | 1.89 (0.51, 7.06) | 0.342 |
| History of fever, days | 219 | 0 | 0.88 (0.75, 1.03) | 0.122 | --- | --- |
| Vomiting, n | 219 | 0 | 3.05 (1.44, 6.48) | 0.004 | 2.98 (1.07, 8.30) | 0.036 |
| Diarrhea, n | 219 | 0 | 2.98 (1.02, 8.76) | 0.047 | 2.67 (0.59, 12.15) | 0.204 |
| Glucose, mmol/L | 209 | 4.6 | 0.80 (0.69, 0.94) | 0.006 | 0.83 (0.68, 1.00) | 0.056 |
| Lactate, mmol/L | 203 | 7.3 | 1.12 (1.02, 1.23) | 0.020 | 1.00 (0.88, 1.14) | 0.944 |
| Platelet count, x10^3^/μL | 217 | 0.9 | 1.00 (0.99, 1.00) | 0.095 | 1.00 (1.00, 1.01) | 0.400 |
| Total bilirubin, mg/dL | 218 | 0.5 | 1.34 (1.12, 1.61) | 0.002 | 1.30 (0.95, 1.78) | 0.103 |
| LDH^1^, u/L | 217 | 0.9 | 9.81 (4.08, 23.59) | <0.0001 | 10.37 (3.24, 33.17) | <0.0001* |
| Plasma HRP2^1^, ng/mL | 219 | 0 | 1.28 (1.02, 1.60) | 0.036 | 1.31 (0.93, 1.85) | 0.122 |

^1^ log(e)transformed

*Significant following Holm’s correction for multiple comparisons (n=11)

## Table S8. AKI and retinopathy using community controls to define AKI

|  | **No AKI (n=137)** | **AKI (n=109)** | **OR (95% CI)** | **P value** | **aOR (95% CI)** | **P value** |
| --- | --- | --- | --- | --- | --- | --- |
| **Retinopathy** | 76 (55.5) | 85 (78.0) | 2.84 (1.62, 5.00) | <0.0001* | 2.04 (1.06, 3.91) | 0.032 |
| Hemorrhages | 60 (43.8) | 74 (68.5) | 2.79 (1.65, 4.74) | <0.0001* | 1.75 (0.95, 3.23) | 0.072 |
| Peripheral whitening | 23 (16.8) | 36 (33.3) | 2.48 (1.36, 4.52) | 0.003* | 2.60 (1.30, 5.21) | 0.007* |
| Macular whitening | 34 (40.5) | 50 (59.5) | 2.61 (1.52, 2.29) | 0.001* | 2.82 (1.49, 5.30) | 0.001* |
| Vessel changes | 32 (23.4) | 27 (25.0) | 1.09 (0.61, 1.97) | 0.765 | 1.01 (0.51, 1.99) | 0.982 |
| Papilloedema | 5 (3.7) | 3 (3.7) | 1.02 (0.27, 3.88) | 0.982 | 0.96 (0.21, 4.46) | 0.961 |
| **Number retinopathy categories^1^**  0  1  2  3  4 | 61 (44.5)  36 (26.3)  19 (13.9)  9 (6.6)  12 (8.8) | 23 (21.3)  31 (28.7)  21 (19.4)  15 (13.9)  18 (16.7) | 2.60 (1.64, 4.14) | <0.0001* | 2.30 (1.36, 3.89) | 0.002* |
| **Severity hemorrhages^2^**  0 (none)  1 (1-5)  2 (6-20)  3 (21-50)  4 (> 50) | 77 (56.2)  32 (23.4)  15 (11.0)  9 (6.6)  4 (2.9) | 34 (31.5)  39 (36.1)  23 (21.3)  6 (6.6)  6 (5.6) | 2.31 (1.44, 3.71) | 0.001* | 1.43 (0.83, 2.45) | 0.196 |

Data presented as n (%) unless otherwise indicated.

Logistic regression used for binary variables and ordinal logistic regression for ordinal variables.

Models adjusted for duration of coma prior to presentation, severe anemia, lactate dehydrogenase and parasite biomass (log(e) transformed).

^1^Retinal hemorrhages, macular whitening, peripheral whitening, vessel changes, ^2^ From worst exam

*Significant following Holm’s adjustment for multiple comparisons (n=8)

## Table S9. AKI and retinopathy using an epidemiologic approach to define AKI

|  | **No AKI (n=161)** | **AKI (n=85)** | **OR (95% CI)** | **P value** | **aOR (95% CI)^3^** | **P value** |
| --- | --- | --- | --- | --- | --- | --- |
| **Retinopathy** | 76 (55.5) | 85 (78.0) | 2.06 (1.15, 3.70) | 0.015 | 1.48 (0.75, 2.90) | 0.255 |
| Hemorrhages | 75 (46.9) | 59 (69.4) | 2.57 (1.47, 4.48) | 0.001* | 1.73 (0.91, 3.27) | 0.094 |
| Peripheral whitening | 32 (20.0) | 27 (31.8) | 1.86 (1.02, 3.39) | 0.042 | 1.73 (0.87, 3.42) | 0.116 |
| Macular whitening | 47 (29.4) | 37 (43.5) | 1.85 (1.07, 3.20) | 0.027 | 1.84 (0.98, 3.45) | 0.058 |
| Vessel changes | 40 (25.0) | 19 (22.4) | 0.86 (0.46, 1.61) | 0.645 | 0.74 (0.36, 1.49) | 0.396 |
| Papilloedema | 5 (3.1) | 4 (4.7) | 1.53 (0.40, 5.86) | 0.531 | 1.65 (0.36, 7.57) | 0.516 |
| **Number retinopathy categories^1^**  0  1  2  3  4 | 64 (40.0)  42 (26.3)  24 (15.0)  13 (8.1)  17 (10.6) | 20 (23.5)  25 (29.4)  16 (18.8)  11 (12.9)  13 (15.3) | 1.86 (1.16, 2.98) | 0.010 | 1.57 (0.92, 2.68) | 0.101 |
| **Severity hemorrhages^2^**  0 (none)  1 (1-5)  2 (6-20)  3 (21-50)  4 (> 50) | 85 (53.1)  41 (25.6)  20 (12.5)  10 (6.3)  4 (2.5) | 26 (30.6)  30 (35.3)  18 (21.2)  5 (5.9)  6 (7.1) | ----- | 0.009 | 1.56 (0.90, 2.69) | 0.114 |

Data presented as n (%) unless otherwise indicated.

Logistic regression used for binary variables and ordinal logistic regression for ordinal variables.

Models adjusted for duration of coma prior to presentation, severe anemia, lactate dehydrogenase and parasite biomass (log(e) transformed)

^1^Retinal hemorrhages, macular whitening, peripheral whitening, vessel changes, ^2^ From worst exam

*Significant following Holm’s adjustment for multiple comparisons (n=8)

## Table S10. Association between AKI, clinical recovery, and survival using community controls to define AKI

|  | **Severe Malarial Anemia (n=219)** | | | **Cerebral Malaria (n=260)** | | |
| --- | --- | --- | --- | --- | --- | --- |
|  | **No AKI** | **AKI** | **P value** | **No AKI** | **AKI** | **P value** |
| **Recovery Times** |  |  |  |  |  |  |
| Fever clearance time, days | 1 (0, 1) | 1 (0, 2) | 0.536 | 1 (1, 2) | 3 (1, 5) | <0.00001* |
| Parasite clearance time, days | 2 (1, 3) | 2 (1, 3) | 0.808 | 2 (1, 3) | 2 (1, 3) | 0.593 |
| Total coma time | ------ | ------ | ------ | 42 (25, 66) | 74 (44, 98) | <0.00001* |
| Duration of hospitalization, days | 3 (2, 4) | 3 (3, 4) | 0.249 | 6 (5, 7) | 7 (6, 9) | 0.0005* |
| **Mortality** |  |  |  |  |  |  |
| In-hospital death, n (%) | 1 (0.6) | 0 (0.0) | 1.000 | 12 (8.2) | 20 (17.7) | 0.020 |
| Post-discharge two year death, n (%) | 4 (2.5) | 5 (9.1) | 0.047 | 0 (0.0) | 2 (2.2) | 0.165 |
| All-cause two year mortality, n (%) | 5 (3.1) | 5 (9.1) | 0.126 | 12 (8.2) | 22 (19.5) | 0.007* |

*Significant following Holm’s correction for multiple comparisons (n=7)

## Table S11. Association between AKI, clinical recovery, and survival using an epidemiologic approach to define AKI

|  | **Severe Malarial Anemia (n=219)** | | | **Cerebral Malaria (n=260)** | | |
| --- | --- | --- | --- | --- | --- | --- |
|  | **No AKI** | **AKI** | **P value** | **No AKI** | **AKI** | **P value** |
| **Recovery Times** |  |  |  |  |  |  |
| Fever clearance time, days | 1 (0, 1) | 1 (0, 2) | 0.582 | 1 (1, 2) | 3 (1, 5) | 0.0001* |
| Parasite clearance time, days | 2 (1, 3) | 2 (1, 3) | 0.424 | 2 (1, 3) | 2 (1, 3) | 0.634 |
| Total coma time, hours | ------ | ------ | ------ | 43 (26, 72) | 76 (46, 97) | <0.0001* |
| Duration of hospitalization, days | 3 (2, 4) | 3 (2, 4) | 0.426 | 6 (5, 7) | 7 (6, 9) | 0.0016* |
| **Mortality** |  |  |  |  |  |  |
| In-hospital death, n (%) | 1 (0.6) | 0 (0.0) | 1.000 | 14 (8.2) | 18 (20.2) | 0.005* |
| Post-discharge 24 month death, n (%) | 6 (3.4) | 3 (7.5) | 0.216 | 0 (0.0) | 2 (2.8) | 0.096 |
| All-cause 24 month mortality, n (%) | 7 (3.9) | 3 (7.5) | 0.395 | 14 (8.2) | 20 (22.5) | 0.001* |

*Significant following Holm’s correction for multiple comparisons (n=7)

## Table S12. Odds ratios of having neurocognitive impairment in children with AKI on hospital admission using community controls to define AKI

|  | OR (95% CI) | P value | Model fit, Pseudo R^2^ | aOR (95% CI)^1^ | P value | Model fit, Pseudo R^2^ |
| --- | --- | --- | --- | --- | --- | --- |
| Baseline | 2.77 (1.78, 4.32) | <0.001* | 0.0407 | 2.31 (1.32, 4.04) | 0.0032* | 0.2444 |
| One year | 3.60 (1.69, 7.65) | 0.001* | 0.0516 | 2.48 (1.01, 6.10) | 0.0482* | 0.2217 |
| Two years | 3.24 (1.54, 6.84) | 0.002* | 0.0438 | 3.03 (1.22, 7.58) | 0.0175* | 0.2259 |

Odds ratio (OR), 95% confidence interval (CI), adjusted OR (aOR). Estimates from logistic regression with neurocognitive impairment as the dependent variable and AKI as the predictor.

^1^ Models adjusted for child age, sex, height-and weight-for age z score, parental education, child education, enrichment in the home environment, disease severity, presence of coma on admission, number of seizures during hospitalization, and parenteral antimalarial treatment (quinine vs. artemisinin-derivative) (n=433 at baseline, n=416 at one year follow-up, n=411 at two years follow-up)

* Significant following Holm’s correction for multiple comparisons (n=3)

## Table S13. Odds ratios of having neurocognitive impairment in children with AKI on hospital admission using an epidemiologic approach to define AKI

|  | OR (95% CI) | P value | Model fit, Pseudo R^2^ | aOR (95% CI)^1^ | P value | Model fit, Pseudo R^2^ |
| --- | --- | --- | --- | --- | --- | --- |
| Baseline | 2.76 (1.73, 4.40) | <0.001* | 0.0357 | 2.54 (1.41, 4.57) | 0.0019* | 0.2463 |
| One year | 2.79 (1.32, 5.88) | 0.007* | 0.0311 | 2.22 (0.91, 5.42) | 0.0793 | 0.2175 |
| Two years | 2.44 (1.15, 5.18) | 0.020* | 0.0231 | 2.42 (0.95, 6.18) | 0.0638 | 0.2153 |

Odds ratio (OR), 95% confidence interval (CI), adjusted OR (aOR). Estimates from logistic regression with neurocognitive impairment as the dependent variable and AKI as the predictor.

^1^ Models adjusted for child age, sex, height-and weight-for age z score, parental education, child education, enrichment in the home environment, disease severity, presence of coma on admission, number of seizures during hospitalization, and parenteral antimalarial treatment (quinine vs. artemisinin-derivative) (n=433 at baseline, n=416 at one year follow-up, n=411 at two years follow-up)

* Significant following Holm’s correction for multiple comparisons (n=3)

**Table S14.**  Relationship between AKI and CKD at follow-up in children exposed to severe malaria

|  | **No CKD (n=350)** | **CKD (n=16)** | **OR (95% CI)** | **P value** |
| --- | --- | --- | --- | --- |
| Community control defined AKI, n (%) | 110 (31.4) | 9 (56.3) | 2.81 (1.02, 7.73) | 0.046* |
| Epidemiologically defined AKI, n (%) | 83 (23.7) | 8 (50.0) | 3.22 (1.17, 8.84) | 0.023* |

* Significant following Holm’s correction for multiple comparisons (n=2)

**References**

1. World Health Organization. Severe Malaria. Trop Med Int Health 2014;19:7-131.

2. Schwartz GJ, Munoz A, Schneider MF, Mak RH, Kaskel F, Warady BA. New equations to estimate GFR in children with CKD. J Am Soc Nephrol 2009;20.

3. Zappitelli M, Parikh CR, Akcan-Arikan A, Washburn KK, Moffett BS, Goldstein SL. Ascertainment and Epidemiology of Acute Kidney Injury Varies with Definition Interpretation. Clinical Journal of the American Society of Nephrology : CJASN 2008;3:948-54.

4. Welch C, Petersen I, Walters K, et al. Two-stage method to remove population- and individual-level outliers from longitudinal data in a primary care database. Pharmacoepidemiology and Drug Safety 2012;21:725-32.

5. Kaufman AS KN. Kaufman Assessment Battery for Children Manual 2nd Edition. Circle Pines, MN: American Guidance Service; 2004.

6. Bangirana P, Seggane M, Allebeck P, et al. A preliminary examination of the construct validity of the KABC-II in Ugandan children with a history of cerebral malaria. African Health Sciences 2009;9:186-92.

7. Bangirana P, Opoka RO, Boivin MJ, et al. Severe Malarial Anemia is Associated With Long-term Neurocognitive Impairment. Clin Infect Dis 2014.

8. Boivin MJ, Bangirana P, Nakasujja N, et al. A year-long caregiver training program improves cognition in preschool Ugandan children with human immunodeficiency virus. J Pediatr 2013;163:1409-16 e1-5.
